# Can the Treatment of Normal-Pressure Hydrocephalus Induce Normal-Tension Glaucoma? A Narrative Review of a Current Knowledge

**DOI:** 10.3390/medicina57030234

**Published:** 2021-03-03

**Authors:** Yasin Hamarat, Laimonas Bartusis, Mantas Deimantavicius, Paulius Lucinskas, Lina Siaudvytyte, Rolandas Zakelis, Alon Harris, Sunu Mathew, Brent Siesky, Ingrida Janulevicienė, Arminas Ragauskas

**Affiliations:** 1 Health Telematics Science Institute, Kaunas University of Technology, K. Barsausko Str. 59-A557, LT-51423 Kaunas, Lithuania; laimonas.bartusis@ktu.lt (L.B.); Mantas.Deimantavicius@ktu.lt (M.D.); Paulius.Lucinskas@ktu.lt (P.L.); rolandas.zakelis@ktu.lt (R.Z.); telematics@ktu.lt (A.R.); 2 Eye Clinic, Lithuanian University of Health Sciences, Eiveniu Str. 2, LT-50009 Kaunas, Lithuania; lynciuke@gmail.com (L.S.); ingrida.januleviciene@kaunoklinikos.lt (I.J.); 3 Glaucoma Research and Diagnostic Center, Eugene and Marilyn Glick Eye Institute, Indiana University School of Medicine, Indianapolis, IN 46202, USA; alharris@indiana.edu (A.H.); sunumath@iupui.edu (S.M.); bsiesky@indiana.edu (B.S.)

**Keywords:** normal-pressure hydrocephalus, normal-tension glaucoma, ventriculoperitoneal shunt, intracranial pressure, lamina cribrosa

## Abstract

Ventriculoperitoneal shunt placement is the most commonly used treatment of normal-pressure hydrocephalus (NPH). It has been hypothesized that normal-tension glaucoma (NTG) is caused by the treatment of NPH by using the shunt to reduce intracranial pressure (ICP). The aim of this study is to review the literature published regarding this hypothesis and to emphasize the need for neuro-ophthalmic follow-up for the concerned patients. The source literature was selected from the results of an online PubMed search, using the keywords “hydrocephalus glaucoma” and “normal-tension glaucoma shunt”. One prospective study on adults, one prospective study on children, two retrospective studies on adults and children, two case reports, three review papers including medical hypotheses, and one prospective study on monkeys were identified. Hypothesis about the association between the treatment of NPH using the shunt to reduce ICP and the development of NTG were supported in all reviewed papers. This suggests that a safe lower limit of ICP for neurological patients, especially shunt-treated NPH patients, should be kept. Thus, we proposed to modify the paradigm of safe upper ICP threshold recommended in neurosurgery and neurology into the paradigm of safe ICP corridor applicable in neurology and ophthalmology, especially for shunt-treated hydrocephalic and glaucoma patients.

## 1. Introduction

Normal-pressure hydrocephalus (NPH) is a neurological disease characterized by enlarged cerebral ventricles and clinical features of gait disturbance, urinary incontinence, and cognitive decline [1,2,3]. Dilation of ventricles is caused by a disturbance in the cerebrospinal fluid (CSF) pathway from production to absorption locations [4]. As the name of this disease suggests, intracranial pressure (ICP) remains in the normal range most of the time [5]. However, the name of normal-pressure hydrocephalus is misleading because continuous monitoring of ICP shows intermittently raised ICP in association with pressure waves [6]. NPH is generally considered to be a disorder of adult and geriatric patients. Around 5.5 patients per 100,000 population undergo ventriculoperitoneal (VP) shunt placement for the treatment of NPH annually, representing one of the commonly performed neurosurgical interventions [7]. VP shunt placement relieves this life-threatening disorder [8], but a hypothesis has recently emerged that treatment of NPH is associated with the development of normal-tension glaucoma (NTG) [9,10].

Glaucoma is a chronic, multifactorial optic nerve (ON) disease characterized by progressive retinal nerve fibers and visual field decline [11]. NTG is a subset of open-angle glaucoma in which intraocular pressure (IOP) is normal (IOP ≤ 21 mmHg), in contrast to high-tension glaucoma (HTG) in which IOP is above 21 mmHg [11,12].

The purpose of this article is to review the literature published regarding the association between the treatment of NPH and the development of NTG, to emphasize the need for neuro-ophthalmic follow-up for patients with shunt-treated NPH.

### 1.1. Pathophysiologic Mechanism of ON Damage in NTG

The pathology of glaucoma may worsen progressively and irreversibly even in cases where IOP, the main risk factor for glaucoma, does not exceed the normal range. Thus, the cause of ON damage in NTG remains a mystery.

It has been suggested that disturbances in ocular blood flow are a major risk factor in the pathogenesis of NTG [13,14]. Vascular complication, such as vasospasms, vasosclerosis, small vessel disease, and autoregulatory dysfunction, leading to perfusion deficits of the ON head, the retina, the choroid or the retrobulbar vessels, might influence the loss of retinal nerve fibers [13,15]. Systemic hypotension, particularly nocturnal arterial hypotension, is another risk factor that is believed to influence the progression of glaucoma [16,17]. Ocular perfusion pressure is defined as the difference between arterial blood pressure (ABP) and IOP. Thus, low ABP causes low ocular perfusion pressure, and this results in ischemic damage to the ON [16]. Yet other pathogenetic factors, such as autoimmunity [18,19], inflammation [20], and accumulation of toxins [21], are believed to contribute to the development of NTG.

**Figure 1:**
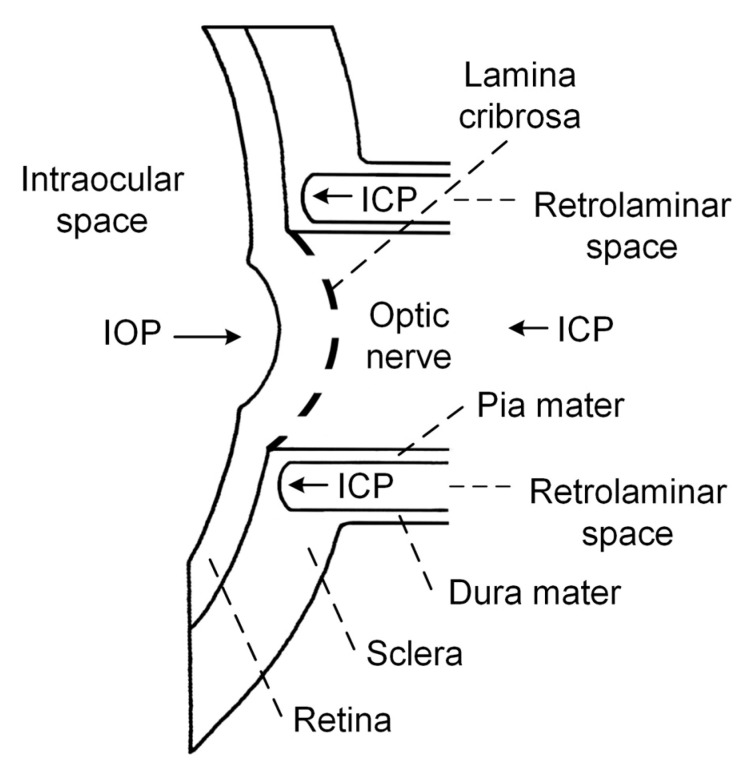
Schematic representation of the relevant intraocular/retrolaminar space. IOP—intraocular pressure, ICP—intracranial pressure.

Currently, attention has been focused on the idea formulated by Volkov back in 1976 that a low ICP may be involved in the pathogenesis of glaucomatous optic neuropathy, because the ON immediately beyond the lamina cribrosa (LC) is surrounded by CSF [22]. Schematic representation of the relevant intraocular/retrolaminar space is depicted in Figure 1 for the explanation of this hypothesis.

The LC is a sieve-like structure, through which the retinal ganglion cell axons pass before forming the ON [23]. The retrolaminar space is a CSF-filled compartment surrounding the ON immediately posterior to the LC [24]. The LC separates the intraocular and retrolaminar spaces and is the possible location of axonal injury.

The pressure change across the LC, called translaminar cribrosa pressure gradient (TCPG), has been proposed to be the primary factor responsible for axonal injury [24,25]. TCPG is calculated as TCPG = (IOP-ICP)/thickness of the LC. According to this hypothesis, TCPG increases due to increased IOP or decreased ICP, and could be responsible for ON damage [26]. In the case of NTG, IOP is within a normal range; therefore, decreases in of ICP might be the cause of ON damage.

The first evidence in favor of this hypothesis appeared in 1979, 3 years after the publication by Volkov. In an experimental study, Yablonski et al. slightly decreased the ICP below the atmospheric pressure by cannulating the cisterna magma in cats [27]. The IOP in one eye was decreased to slightly exceed atmospheric pressure by cannulation of the anterior chamber, whereas the remaining eye was left untouched. After 3 weeks, the optic disc of the eye in which IOP was unchanged showed glaucomatous optic neuropathy; in contrast, no sign of glaucoma was identified in the eye that had reduced IOP. Since then, little attention has been focused on the role of ICP in the development of glaucoma until recent years.

In a retrospective study, Berdahl et al. reviewed the medical records of 62,468 patients who had lumbar puncture readings [28]. These authors selected 57 open-angle glaucoma patients (including 11 NTG patients) and 105 age-matched control subjects (patients with no signs of glaucoma) for the comparison of ICP. The mean ICP was significantly lower in open-angle glaucoma patients (9.6 ± 3.1 mmHg) and NTG patients (9.3 ± 3.2 mmHg) compared with control subjects (12.7 ± 3.9 mmHg).

In a prospective study, Ren et al. measured ICP via lumbar puncture on 29 HTG patients, 14 NTG patients and 71 subjects without glaucoma [29]. The mean ICP was significantly lower in NTG patients (9.5 ± 2.2 mmHg) than in the control subjects (12.9 ± 1.9 mmHg) and HTG group (11.7 ± 2.7 mmHg).

In a prospective pilot study, Siaudvytyte and colleagues measured ICP using a novel two-depth transcranial Doppler (TCD) based non-invasive ICP measurement device, including nine patients with NTG, nine patients with HTG, and nine healthy controls [30]. The mean ICP was found to be lower in NTG patients (7.4 ± 2.7 mmHg) than healthy controls (10.5 ± 3.0 mmHg) and the HTG group (8.9 ± 1.9 mmHg), although differences between the groups were not statistically significant.

In a most recent prospective study, conducted by Linden and colleagues, ICP was measured via lumbar puncture by using the Likvor CELDA system on 13 NTG patients and 51 healthy volunteers [31]. The mean ICP measured in a supine body position was lower in the NTG patients (10.3 ± 2.7 mmHg) than healthy volunteers (11.3 ± 2.2 mmHg). However, differences between the groups were not statistically significant.

Although all four studies showed lower mean ICP values measured on NTG patients compared with control subjects, a final conclusion in favor of the proposed hypothesis cannot yet be stated. The largest study was retrospective, and the remaining three, although prospective, did not enroll a large group of NTG patients and could be titled pilot studies.

Dysfunction of an occlusion mechanism of the ON sheath around the ON has been proposed recently as a pathophysiologic component in NTG [32]. The unifying glymphatic hypothesis of glaucoma, which incorporates vascular, biomechanical, and biochemical factors to explain the pathophysiology of glaucomatous optic neuropathy, has also been proposed [25,33].

### 1.2. Association between NPH and NTG

The possible role of decreased ICP in the development of NTG is relevant to the treatment of NPH. Guidelines for the management of NPH indicate that the most effective treatment is surgical procedures during which either ventriculoperitoneal, ventriculoatrial, or lumboperitoneal shunts are implanted [34]. According to structural features, the shunt systems are categorized into four groups: fixed differential pressure valves, programmable valves, gravity-assisted valves, and flow-regulated valves. Currently, there is no generally established method for setting the initial valve pressure value. Low pressure (0.4–3.7 mmHg) or medium pressure (3.8–8.1 mmHg) fixed differential pressure valves have been recommended and used [34]. Reference tables to set the initial pressure value of the programmable valves based on the patient’s height and weight have been suggested [34,35]. These pressure values range from 2.2 mmHg to 14.7 mmHg for males and females. Mean ICP values measured on NTG patients (9.3 ± 3.2 mmHg [28], 9.5 ± 2.2 mmHg [29], 7.4 ± 2.7 mmHg [30], 10.3 ± 2.7 mmHg [31]) are even higher compared to recommended initial pressure values of valves used to treat NPH patients.

A question can be raised as follows: does the treatment of NPH by using the shunt to reduce ICP cause NTG? To find the answer, we have performed an online literature search in the database PubMed using the following search terms: “hydrocephalus glaucoma”, “normal-tension glaucoma shunt”. The references and citation indices of the selected articles were hand-searched for additional relevant articles. Papers have been included in this review after first screening the titles and later reading relevant abstracts with whole articles. One prospective study on adults, one prospective study on children, two retrospective studies on adults and children, two case reports, three review papers, and one prospective study on monkeys were found to be relevant to the question of this paper. A structured comparative description of the studies is depicted in Table 1.

### 1.3. Prospective Studies

The prevalence of NTG in patients with NPH who had implanted VP shunt for the treatment was estimated by Gallina et al. [26]. The extent of the ON exposure (time between the shunt placement and ophthalmic examination) to the change of the pressure difference IOP-ICP in relation to NTG occurrence was also evaluated in this study. Nine of 22 patients had NTG, which is 40-fold more compared with the general elderly population without NPH. The authors concluded that the main risk factor for the development of NTG in shunt-treated NPH patients is the duration of ON exposure to the lowering of ICP. According to the authors of this study, the next step should be the determination of tolerated times for a given ICP decrease.

Retinal nerve fiber layer (RNFL) thickness and neuroretinal rim area (NRA) of the ON head were examined after reduction of ICP on Rhesus monkeys [36]. Lumbar-peritoneal CSF shunt was implanted in nine monkeys. The shunt was opened to achieve roughly 3 mmHg of ICP in four monkeys, while the shunt was left closed in five monkeys. A follow-up of 1 year was done by taking optic coherence tomography and photographic images of the ON head and RNFL of all monkeys. At the beginning of the study, RNFL thickness and NRA did not differ significantly between groups of monkeys with an opened shunt and closed shunt. During a follow-up, two monkeys with an opened shunt (50%) demonstrated a progressive reduction in RNFL thickness in both of their eyes, followed by a significant reduction in the NRA. An optic disc hemorrhage was identified at the fourth month after the beginning of the study in the right eye of the third monkey with an opened shunt. Mean RNFL thickness was significantly less in the monkeys with opened shunt 89.4 ± 15 µm than in the monkeys with closed shunt 100.9 ± 7.4 µm at the end of the study. The authors of this study concluded that their findings support the concept that a low ICP might be a risk factor in all forms of optic neuropathy, including glaucoma.

Visual field examination has been performed in children with shunt-treated hydrocephalus by Rudolph et al. [37]. A total of 56 ophthalmological examinations were performed on 32 boys and 24 girls. Visual field testing was possible in 44 cases. The examination was incomplete in 12 patients with cognitive deficits or inadequate compliance. Visual field deficits were observed in 24 patients which is 54.5% of all completed examinations. There were visual field constrictions between 10 degrees and 50 degrees out of the center. The authors concluded that children with shunt-treated hydrocephalus have an increased risk of ophthalmological abnormalities and should be more intensively ophthalmologically monitored.

### 1.4. Retrospective Studies

Electronic medical records of 72 NPH patients and 72 controls were retrospectively analyzed by Chang and Singh to test the hypothesis that the prevalence of glaucoma is higher in patients with NPH compared to adult age-matched and race-matched controls [38]. These authors estimated that the prevalence of glaucoma in NPH was 18.1% (13 cases) in contrast to 5.6% (four cases) in controls. Authors of this retrospective study concluded that the three-fold greater likelihood of glaucoma diagnosis in the NPH group is a significant finding that deserves further prospective study. However, the number of NTG cases was unknown. Thus, the prevalence of NTG in NPH patients was not determined. Also, the distinction between shunt-treated and non-shunt treated patients was not performed, which made it impossible to separate cases of glaucoma associated with CSF diversion.

Medical records of 137 shunted hydrocephalic children patients were retrospectively reviewed by Heinsbergen et al. to identify the main risk factors for outcome [39]. The type and prevalence of impairment, including visual function, was also listed. One-hundred-nineteen patients were included in the analysis. The diagnosis of hydrocephalus was made in 90% of the patients before the age of 1 year and in rest after 1 year of age. The median age at the assessment of the outcome was 5 years. It was found that 25% of all patients were diagnosed with visual function impairment.

### 1.5. Case Reports

NTG was diagnosed in a 93-year-old white woman in May 2000 [40]. A VP shunt with a programmable valve set at 8.8 mmHg had been placed in this patient for the treatment of NPH in September 2011. Two optic disc hemorrhages were detected in the right eye and worsening of visual fields in both eyes was noted 1 month after implantation of the VP shunt. The pressure value of the programmable valve was increased up to 14.7 mmHg. After this, ophthalmic examination showed that disc hemorrhages in the right eye had disappeared. Pressure setting of the VP shunt was decreased multiple times because the patient developed signs of NPH; first, from 14.7 to 13.2 mmHg in November 2011; second, from 13.2 to 11.8 mmHg in December 2011; third, from 11.8 to 11.0 mmHg in January 2012; and fourth, from 11.0 to 10.3 mmHg in February 2012. A new optic disc hemorrhage was detected in the right eye, and again worsening of visual fields in both eyes was noted in August 2012. Optic disc hemorrhage was identified in the left eye in September 2013. At this time, the pressure setting of the VP shunt was increased to 11.0 mmHg. No disc hemorrhages were visible in November 2013. The authors concluded that this case is the first report of worsening of NTG obviously caused by lowering the ICP after implantation of the VP shunt for the treatment of NPH.

A 2-year-old boy developed hydrocephalus after the treatment of malignant pineoblastoma [41]. VP, ventriculopleural, and ventriculoatrial shunts were placed in succession for the treatment of hydrocephalus. The patient underwent eight shunt revisions in a period of 25 years, during which ICP values below the normal range according to the age of the patient have been identified at last six revisions. A diagnosis of juvenile-onset NTG was made in his only seeing left eye after detailed ophthalmic examinations. A programmable shunt has been implanted in the patient at the age of 27 years to relieve headaches and reduce the risk of progressive glaucomatous visual loss. Four weeks after the implantation, the ICP had stabilized at 6 to 13 mmHg, which approximately falls into the normal ICP range at that age (8 to 15 mmHg). The authors concluded that this case describes a human model of juvenile-onset NTG caused by low ICP, in which all other non-IOP-dependent pathophysiological mechanisms have been excluded.

### 1.6. Review Papers Including Medical Hypotheses

Bokhari and Baeesa described the clinical and theoretical basis for their hypothesis after reviewing literature regarding the increased prevalence of glaucoma among NPH patients treated using a CSF diversion procedure [9]. The authors hypothesized that besides the recently included pathophysiologic mechanism of TCPG increase in the pathogenesis of NTG, inherently fragile neurons in NPH patients could be another possible reason for an increased prevalence of glaucoma. The authors even suggested including TCPG into the treatment guidelines of NPH. They expect that recent advances in the imaging of the ON head complex might provide an opportunity to detect the mechanical changes of an increased TCPG before ON damage happened.

Wostyna et al. have reviewed the literature and discussed low ICP as a risk factor for the development of NTG [42]. As the authors of this paper revealed growing evidence of the important role of low ICP in the pathogenesis of glaucoma, including the development of NTG after treatment of NPH, they hypothesized that in the future, glaucoma could be treated from the intracranial compartment side of the LC. The authors proposed an implantable CSF pump—an invasive system to infuse artificial CSF into the intrathecal space surrounding the spinal cord, thus increasing the ICP—as a novel strategy for the treatment of glaucoma.

McCulley et al. summarized the published works regarding relationships between ICP and glaucoma and the current evidence supporting or denying ICP as a risk factor for glaucoma [24]. Association between NTG and treatment of NPH by the CSF shunting procedure that reduces ICP was also discussed. The authors concluded that already published data support the notion that low ICP is a risk factor for glaucoma and might be responsible for the increased rate of NTG observed in NPH patients.

## 2. Results

The hypothesis about the association between the treatment of NPH using a shunt to reduce ICP and development of NTG is supported in all above-reviewed retrospective studies, case reports, and review papers. However, these works do not belong to the first-level of evidence in evidence-based medicine. Three prospective studies—with first-level evidence, although all including relatively small sample size (22 adult humans [26], 44 children [37], 9 Rhesus monkeys [36])—also revealed a convincing link between the reduction of ICP and development of NTG. Summarizing all of these results, it seems that a safe lower limit of ICP for neurological patients, especially shunt-treated NPH patients, should be retained. If so, the paradigm of a safe upper ICP threshold (as recommended in neurosurgery ICP < 20 mmHg [43,44] or ICP < 22 mmHg [44,45], and neurology ICP < 14.7 mmHg [46,47]) should be modified into the paradigm of safe ICP corridor applicable in neurology and ophthalmology, especially for shunt-treated hydrocephalic and glaucoma patients, to avoid progression of NTG.

Although the ICP upper threshold values used to initiate treatment in neurosurgery were recommended many years ago, these are still debatable [44]. Whereas no data are available to formulate recommendations of lower ICP thresholds used to minimize or eliminate the risk of NTG development. There is no well-established technique to set initial postoperative valve pressure value. Reference tables to set the initial pressure value of programmable valves based on the patient’s height and weight have been suggested without considering the risk of NTG development [34,35]. These pressure values range from 2.2 mmHg to 14.7 mmHg, both for males and females. Postoperative measurement of ICP using an invasive external ventricular drain or an intraparenchymal monitor is undesirable for shunt-treated patients because of a too high risk of infection, restricted mobility, and short-term use, among other reasons [48]. Most of the time, anamnesis, clinical examination, imaging studies, and the physician’s judgment are used to adjust valve settings for proper CSF drainage. However, measurement of ICP would be valuable in postoperative shunt complications, shunt occlusion, infection, headache, and subdural hematoma, among other scenarios.

Gallina et al. suggested not searching for a threshold of ICP itself, but a threshold of IOP–ICP difference (ΔP) multiplied by exposure time (t) of such a difference, because they found that duration of ON exposure to the lowering of ICP is very influential for the development of NTG in shunt-treated NPH patients [26]. First-level evidence studies will be needed to derive these threshold values.

While IOP measurement, although indirect, is non-invasive and easily obtained by using an applanation tonometer, ICP measurement is complicated because invasive procedures, which can be done by a neurosurgeon, are needed to obtain a reliable ICP value. ICP measurement by non-invasive means would enable better postoperative management of shunt-treated patients and would encourage future studies for the search of safe lower ICP or upper ΔP×t threshold values. Plenty of the suggested non-invasive ICP assessment approaches, such as measurement of the pulsatility index in the middle cerebral artery, fundoscopy, measurement of the optic nerve sheath diameter, magnetic resonance imaging and assessment of tympanic membrane displacement, are unable to measure absolute ICP value accurately and have not been used in clinical studies for the NTG diagnosis [49,50]. We identified only one (recently published) study on glaucoma patients in which ICP has been measured in a non-invasive manner [30]. A promising two-depth TCD device has been used for the non-invasive measurement of ICP in this prospective pilot study. The accuracy, precision, diagnostic sensitivity, and specificity of this non-invasive ICP technology have been independently tested on neurological patients by various groups [51,52,53]. Although, the results showed fair agreement to invasively measured ICP with normal, high, or slightly elevated ICP in neurological patients, there is no accurate validation of this technology in a lower range of ICP (0–8 mmHg) tested in NTG patients.

Future advances in two-depth TCD non-invasive ICP measurement technology or other should allow searching for safe lower ICP thresholds which would enable NPH treatment without or with minimal risk of NTG development and would put the paradigm of a safe ICP corridor into clinical practice.

While ICP has recently been considered to influence the development of glaucoma, yet another physiological mechanism also from the intracranial compartment, cerebrovascular autoregulation (CA), started to emerge as a factor that might play a role in the pathogenesis of glaucomatous optic neuropathy [54,55].

## 3. Conclusions

The available data suggest that there is a link between the treatment of NPH using a shunt to reduce ICP and the development of NTG. Unfortunately, there are no published data on a safe lower ICP patient-specific threshold, which would help to improve NPH patients while the risk of NTG development would be minimal. Definitively validated non-invasive measurement of ICP would encourage searching for such threshold values.

A limited amount of data also suggests that CA might play a role in the pathogenesis of glaucomatous optic neuropathy. Prospective CA monitoring studies on NPH patients who have a risk of NTG development are now needed.

## Figures and Tables

**Table 1:** Summary of those studies analyzing the association between the treatment of normal-pressure hydrocephalus using a shunt to reduce intracranial pressure and development of normal-tension glaucoma. Review papers are not included in this table.

Study	Method	Sample Size	Age, Years	Study Group	Control Group	Time Frame	Glaucomatous Damage
Gallina P, et al. [26]	Prospective study	12 males, 10 females	Range of 68–87 years	22 adult	-	6 months	9 patients
Yang D, et al. [36]	Prospective study	9 male Rhesus monkeys	An average age of 6 years	4 Rhesus monkeys	5 Rhesus monkeys	12 months	3 Rhesus monkeys
Rudolph D, et al. [37]	Prospective study	32 boys, 24 girls	An average age of 15 years	56 children	-	12 months	13 patients
Chang TC and Singh K. [38].	Retrospective study	67 males, 77 females	An average age of 75 years	72 adult	72 adult	132 months	13 patients
Heinsbergen I, et al. [39]	Retrospective study	67 males, 52 females	Range of 1–5 years	119 children	-	96 months	N/S
Chen BH, et al. [40]	Case report	1 female	93	1 adult	-	162 months	1 patient
Yusuf IH, et al. [41]	Case report	1 male	27	1 adult	-	300 months	1 patient

N/S: not specified.

## References

[1] Kotagal V., Walkowiak E., Heth J.A. (2018). Serious adverse events following Normal Pressure Hydrocephalus surgery. Clin. Neurol. Neurosurg..

[2] Lalou A.D., Czosnyka M., Donnelly J., Pickard J.D., Nabbanja E., Keong N.C., Garnett M., Czosnyka Z.H. (2018). Cerebral autoregulation, cerebrospinal fluid outflow resistance, and outcome following cerebrospinal fluid diversion in normal pressure hydrocephalus. J. Neurosurg. JNS.

[3] Shprecher D., Schwalb J., Kurlan R. (2008). Normal pressure hydrocephalus: Diagnosis and treatment. Curr. Neurol. Neurosci. Rep..

[4] Gholampour S. (2018). FSI simulation of CSF hydrodynamic changes in a large population of non-communicating hydrocephalus patients during treatment process with regard to their clinical symptoms. PLoS ONE.

[5] Czosnyka Z., Czosnyka M. (2017). Long-term monitoring of intracranial pressure in normal pressure hydrocephalus and other CSF disorders. Acta Neurochir. (Wien.).

[6] Wostyn P., Audenaert K., De Deyn P.P. (2008). Alzheimer’s disease-related changes in diseases characterized by elevation of intracranial or intraocular pressure. Clin. Neurol. Neurosurg..

[7] Agarwal N., Kashkoush A., McDowell M.M., Lariviere W.R., Ismail N., Friedlander R.M. (2018). Comparative durability and costs analysis of ventricular shunts. J. Neurosurg. JNS.

[8] Paulsen A.H., Due-Tønnessen B.J., Lundar T., Lindegaard K.-F. (2017). Cerebrospinal fluid (CSF) shunting and ventriculocisternostomy (ETV) in 400 pediatric patients. Shifts in understanding, diagnostics, case-mix, and surgical management during half a century. Child’s Nerv. Syst..

[9] Bokhari R.F., Baeesa S.S. (2013). Does the treatment of normal pressure hydrocephalus put the retinal ganglion cells at risk? A brief literature review and novel hypothesis. Med. Hypotheses.

[10] Wostyn P., Audenaert K., De Deyn P.P. (2010). High Occurrence Rate of Glaucoma Among Patients with Normal Pressure Hydrocephalus. J. Glaucoma.

[11] Pircher A., Montali M., Wostyn P., Pircher J., Berberat J., Remonda L., Killer H.E. (2018). Impaired cerebrospinal fluid dynamics along the entire optic nerve in normal-tension glaucoma. Acta Ophthalmol..

[12] Xu H., Zhai R., Zong Y., Kong X., Jiang C., Sun X., He Y., Li X. (2018). Comparison of retinal microvascular changes in eyes with high-tension glaucoma or normal-tension glaucoma: A quantitative optic coherence tomography angiographic study. Graefe’s Arch. Clin. Exp. Ophthalmol. = Albr. von Graefes Arch. fur Klin. Exp. Ophthalmol..

[13] Plange N., Remky A., Arend O. (2003). Colour Doppler imaging and fluorescein filling defects of the optic disc in normal tension glaucoma. Br. J. Ophthalmol..

[14] Sugiyama T., Utsunomiya K., Ota H., Ogura Y., Narabayashi I., Ikeda T. (2006). Comparative study of cerebral blood flow in patients with normal-tension glaucoma and control subjects. Am. J. Ophthalmol..

[15] Fan N., Wang P., Tang L., Liu X. (2015). Ocular Blood Flow and Normal Tension Glaucoma. Biomed Res. Int..

[16] Levine R.M., Yang A., Brahma V., Martone J.F. (2017). Management of Blood Pressure in Patients with Glaucoma. Curr. Cardiol. Rep..

[17] Hayreh S.S., Zimmerman M.B., Podhajsky P., Alward W.L. (1994). Nocturnal arterial hypotension and its role in optic nerve head and ocular ischemic disorders. Am. J. Ophthalmol..

[18] Grus F.H., Joachim S.C., Wuenschig D., Rieck J., Pfeiffer N. (2008). Autoimmunity and glaucoma. J. Glaucoma.

[19] Wax M.B. (2011). The case for autoimmunity in glaucoma. Exp. Eye Res..

[20] Vohra R., Tsai J.C., Kolko M. (2013). The role of inflammation in the pathogenesis of glaucoma. Surv. Ophthalmol..

[21] Lee S.H., Kang E.M., Kim G.A., Kwak S.W., Kim J.M., Bae H.W., Seong G.J., Kim C.Y. (2016). Three Toxic Heavy Metals in Open-Angle Glaucoma with Low-Teen and High-Teen Intraocular Pressure: A Cross-Sectional Study from South Korea. PLoS ONE.

[22] Volkov V. (1976). V Essential element of the glaucomatous process neglected in clinical practice. Oftalmol. Zh..

[23] Morgan-Davies J., Taylor N., Hill A.R., Aspinall P., O’Brien C.J., Azuara-Blanco A. (2004). Three dimensional analysis of the lamina cribrosa in glaucoma. Br. J. Ophthalmol..

[24] McCulley T.J., Chang J.R., Piluek W.J. (2015). Intracranial pressure and glaucoma. J. Neuro-Ophthalmol. Off. J. N. Am. Neuro-Ophthalmol. Soc..

[25] Wostyn P., Van Dam D., Audenaert K., Killer H.E., De Deyn P.P., De Groot V. (2015). A new glaucoma hypothesis: A role of glymphatic system dysfunction. Fluids Barriers CNS.

[26] Gallina P., Savastano A., Becattini E., Orlandini S., Scollato A., Rizzo S., Carreras G., Di Lorenzo N., Porfirio B. (2017). Glaucoma in patients with shunt-treated normal pressure hydrocephalus. J. Neurosurg. JNS.

[27] Yablonski M.E., Ritch R., Pokorny K.S. (1979). Effect of decreased intracranial-pressure on optic disk. Investigative Ophthalmology & Visual Science.

[28] Berdahl J.P., Fautsch M.P., Stinnett S.S., Allingham R.R. (2008). Intracranial pressure in primary open angle glaucoma, normal tension glaucoma, and ocular hypertension: A case-control study. Investig. Ophthalmol. Vis. Sci..

[29] Ren R., Jonas J.B., Tian G., Zhen Y., Ma K., Li S., Wang H., Li B., Zhang X., Wang N. (2010). Cerebrospinal Fluid Pressure in Glaucoma. A Prospective Study. Ophthalmology.

[30] Siaudvytyte L., Januleviciene I., Ragauskas A., Bartusis L., Meiliuniene I., Siesky B., Harris A. (2014). The difference in translaminar pressure gradient and neuroretinal rim area in glaucoma and healthy subjects. J. Ophthalmol..

[31] Lindén C., Qvarlander S., Jóhannesson G., Johansson E., Östlund F., Malm J., Eklund A. (2018). Normal-Tension Glaucoma Has Normal Intracranial Pressure: A Prospective Study of Intracranial Pressure and Intraocular Pressure in Different Body Positions. Ophthalmology.

[32] Jóhannesson G., Eklund A., Lindén C. (2018). Intracranial and Intraocular Pressure at the Lamina Cribrosa: Gradient Effects. Curr. Neurol. Neurosci. Rep..

[33] Wostyn P., De Groot V., Van Dam D., Audenaert K., Killer H.E., De Deyn P.P. (2017). The Glymphatic Hypothesis of Glaucoma: A Unifying Concept Incorporating Vascular, Biomechanical, and Biochemical Aspects of the Disease. Biomed. Res. Int..

[34] Mori E., Ishikawa M., Kato T., Kazui H., Miyake H., Miyajima M., Nakajima M., Hashimoto M., Kuriyama N., Tokuda T. (2012). Guidelines for management of idiopathic normal pressure hydrocephalus: Second edition. Neurol. Med. Chir..

[35] Miyake H., Kajimoto Y., Tsuji M., Ukita T., Tucker A., Ohmura T. (2008). Development of a Quick Reference Table for Setting Programmable Pressure Valves in Patients With Idiopathic Normal Pressure Hydrocephalus. Neurol. Med. Chir..

[36] Yang D., Fu J., Hou R., Liu K., Jonas J.B., Wang H., Chen W., Li Z., Sang J., Zhang Z. (2014). Optic neuropathy induced by experimentally reduced cerebrospinal fluid pressure in monkeys. Invest. Ophthalmol. Vis. Sci..

[37] Rudolph D., Sterker I., Graefe G., Till H., Ulrich A., Geyer C. (2010). Visual field constriction in children with shunt-treated hydrocephalus. J. Neurosurg. Pediatr..

[38] Chang T.C., Singh K. (2009). Glaucomatous disease in patients with normal pressure hydrocephalus. J. Glaucoma.

[39] Heinsbergen I., Rotteveel J., Roeleveld N., Grotenhuis A. (2002). Outcome in shunted hydrocephalic children. Eur. J. Paediatr. Neurol. EJPN Off. J. Eur. Paediatr. Neurol. Soc..

[40] Chen B.H., Drucker M.D., Louis K.M., Richards D.W. (2016). Progression of Normal-Tension Glaucoma After Ventriculoperitoneal Shunt to Decrease Cerebrospinal Fluid Pressure. J. Glaucoma.

[41] Yusuf I.H., Ratnarajan G., Kerr R.S., Salmon J.F. (2016). Juvenile-onset Normal Tension Glaucoma From Chronic, Recurrent Low Cerebrospinal Fluid Pressure. J. Glaucoma.

[42] Wostyn P., Van Dam D., De Deyn P.P. (2018). Intracranial pressure and glaucoma: Is there a new therapeutic perspective on the horizon?. Med. Hypotheses.

[43] Honda M., Ichibayashi R., Suzuki G., Yokomuro H., Seiki Y., Sase S., Kishi T. (2017). Consideration of the Intracranial Pressure Threshold Value for the Initiation of Traumatic Brain Injury Treatment: A Xenon CT and Perfusion CT Study. Neurocrit. Care.

[44] Stocchetti N., Poole D., Okonkwo D.O. (2018). Intracranial pressure thresholds in severe traumatic brain injury: We are not sure: Prudent clinical practice despite dogma or nihilism. Intensive Care Med..

[45] Carney N., Totten A.M., O’Reilly C., Ullman J.S., Hawryluk G.W.J., Bell M.J., Bratton S.L., Chesnut R., Harris O.A., Kissoon N. (2017). Guidelines for the Management of Severe Traumatic Brain Injury, Fourth Edition. Neurosurgery.

[46] Ropper A.H., Brown R.H. (2005). Adams and Victor’s Principles of Neurology.

[47] Czosnyka M., Pickard J.D. (2004). Monitoring and interpretation of intracranial pressure. J. Neurol. Neurosurg. Psychiatry.

[48] Antes S., Stadie A., Müller S., Linsler S., Breuskin D., Oertel J. (2018). Intracranial Pressure-Guided Shunt Valve Adjustments with the Miethke Sensor Reservoir. World Neurosurg..

[49] Raboel P.H., Bartek J.J., Andresen M., Bellander B.M., Romner B. (2012). Intracranial Pressure Monitoring: Invasive versus Non-Invasive Methods-A Review. Crit. Care Res. Pract..

[50] Siaudvytyte L., Januleviciene I., Ragauskas A., Bartusis L., Siesky B., Harris A. (2015). Update in intracranial pressure evaluation methods and translaminar pressure gradient role in glaucoma. Acta Ophthalmol..

[51] Ragauskas A., Matijosaitis V., Zakelis R., Petrikonis K., Rastenyte D., Piper I., Daubaris G. (2012). Clinical assessment of noninvasive intracranial pressure absolute value measurement method. Neurology.

[52] Ragauskas A., Bartusis L., Piper I., Zakelis R., Matijosaitis V., Petrikonis K., Rastenyte D. (2014). Improved diagnostic value of a TCD-based non-invasive ICP measurement method compared with the sonographic ONSD method for detecting elevated intracranial pressure. Neurol. Res..

[53] Bershad E.M., Anand A., DeSantis S.M., Yang M., Tang R.A., Calvillo E., Malkin-Gosdin L., Foroozan R., Damani R., Maldonado N. (2016). Clinical Validation of a Transcranial Doppler-Based Noninvasive Intracranial Pressure Meter: A Prospective Cross-Sectional Study. World Neurosurg..

[54] Harris A., Siesky B., Wirostko B. (2013). Cerebral blood flow in glaucoma patients. J. Glaucoma.

[55] Kumpaitiene B., Svagzdiene M., Sirvinskas E., Adomaitiene V., Petkus V., Zakelis R., Krakauskaite S., Chomskis R., Ragauskas A., Benetis R. (2019). Cerebrovascular autoregulation impairments during cardiac surgery with cardiopulmonary bypass are related to postoperative cognitive deterioration: Prospective observational study. Minerva Anestesiol..

